# Operative and non-operative treatment of symptomatic adult spinal deformity: a prospective multicenter observational study

**DOI:** 10.1186/s12891-026-10232-1

**Published:** 2026-07-24

**Authors:** Vinjar Brenna Hansen, Bungo Otsuki, Freyr Gauti Sigmundsson, Lien My Diep, Thomas Kibsgård, Stephan M. Röhrl, Christian Hellum

**Affiliations:** 1 Department of Clinical Medicine University of Bergen, Jonas Lies vei 91 B, Bergen, 5021 Norway; 2 Orthopedic Department, Oslo University Hospital, Rikshospitalet, Pb4950 Nydalen, Oslo, 0424 Norway; 3 Orthopedic Department, Oslo University Hospital, Ullevål, Pb4956 Nydalen, Oslo, 0424 Norway; 4 OCBE, Research Support Services, Oslo University Hospital, Oslo, Norway; 5 Institute for Clinical Medicine, University of Oslo, Pb 1171 Blindern, Oslo, 0381 Norway; 6 Orthopedic Department, Kyoto University Hospital, 54 Shogoin Kawaracho, Kyoto, Japan; 7 Orthopedic Department, Örebro University Hospital, Södra Grev Rosengatan 42, Örebro, S-703 62 Sweden

**Keywords:** Adult spinal deformity, Spine surgery, Non-surgical treatment, Oswestry Disability Index, Prospective multicenter study, Patient-reported outcome measures (PROMs)

## Abstract

**Purpose:**

Few prospective studies describe surgical treatment (ST) and non-surgical treatment (NST) of symptomatic adult spinal deformity (ASD). In this study, we aimed to describe short term outcomes following ST and to evaluate the feasibility and clinical course of a structured NST strategy in patients with symptomatic adult spinal deformity.

**Methods:**

This is an observational multicenter study conducted in Norway, Sweden, and Japan. According to the study criteria, patients with symptomatic ASD were selected at outpatient clinics. The responsible surgeon and the patient decided on either surgery at the surgeon’s discretion or a non-surgical procedure guided by a physiotherapist. Oswestry Disability Index (ODI) was the primary outcome. Secondary outcomes included success defined as a ≥ 30% improvement in ODI score, failure (≥ 10% worsening in ODI), SRS-22, the numeric rating scale (NRS) for back and leg pain, and reoperation.

**Results:**

We followed 76 ST and 16 NST patients for up to 12 months with no patients crossing over. A statistically significant improvement in ODI was only observed amongst the ST patients. The secondary outcomes measures aligned with the primary outcomes. Successful treatment (≥ 30% improvement in ODI) was achieved in 37 of 68 (54.4%), and nine out of 68 (13.2%) experienced treatment failure (≥ 10% worsening in ODI). Revision rate was 3/76 (3.9%) within 12 months. Among the 16 non-surgical patients, 1/16 (6.3%) achieved a successful result. The failure rate was 8/16 (50%) due to pain limiting the feasibility of the specific exercise protocol. Again, the secondary outcomes aligned with the primary outcomes.

**Conclusion:**

A statistically significant improvement in ODI was only observed amongst the ST patients. Among the surgical patients, just over half experienced successful treatment, defined as a ≥ 30% improvement in ODI. Under the present implementation and patient selection, NST was associated with limited clinical improvement and substantial feasibility constraints due to pain, with patients, on average, experiencing a deterioration in their symptoms. This suggests that feasibility and tolerability, rather than intrinsic inefficacy of core strengthening, may explain the limited short term clinical improvement observed. The results primarily describe short-term outcomes following ST and the feasibility-limited implementation of the NST protocol, while between-group comparison should be considered exploratory.

**Trial registration:**

Indication and Treatment of Adult Kyphoscoliosis (INTRAKS) Clinical Trials ID NCT04536909, protocol ID 1126, 27/04/2020. Retrospectively registered inclusion started in January 2020.

**Supplementary Information:**

The online version contains supplementary material available at https://doi.org/10.1186/s12891-026-10232-1.

## Introduction

Surgical treatment (ST) for adult spinal deformity (ASD) often yields mediocre outcomes and carries a significant risk of complications [1–3]. Adult deformity surgery (ADS) encompasses a broad spectrum of pathologies and indications, including degenerative kyphosis, scoliosis, ankylosing spondylitis, and post-fracture sequelae [2, 4, 5]. Despite this variety, outcomes, complications, and rates of reoperation are consistent across different conditions and surgical procedures [2–4]. Given the high incidence of complications and necessity for reoperation, non-surgical treatment (NST) may appear more appealing.

There are few prospective studies for NST in ASD patients, and, to our knowledge, none include a specified core strengthening and trunk stabilizing program [1, 6–8]. ST is associated with complications, reoperations, and health care costs [2, 9]. Consequently, alternative conservative treatment strategies or options should be sought. For less severe spinal pathology, training positively impacts patient satisfaction and outcomes [10]. The available NST studies include non-specific physical exercise, analgesia, and facet joint and perineural infiltration [1, 7]. In these studies, most patients had no benefit from non-surgical regimes in measured health-related quality of life (HRQOL), pain, or disability [1, 7]. To our knowledge, most published studies lack uniformity and a detailed exercise strategy [11, 12].

The aim of this study was not to establish comparative effectiveness, but to describe short-term outcome following ST and to explore the feasibility and clinical course of a structured NST strategy in patients with adult spinal deformity.

## Materials and methods

All patients signed an informed consent form. Data was collected through the study homepage (intraks.org) using online forms (nettskjema.no) and stored in “Services for sensitive data” (TSD) at the University of Oslo.

### Patients

Patients were enrolled at Oslo University Hospital and Haukeland University Hospital in Norway, Örebro University Hospital in Sweden and Kyoto University Hospital in Japan (January 2020 – December 2023). All evaluated patients were potential surgical candidates, and patients were allocated to surgical or non-surgical treatment through shared decision making. Non-surgical patients were only included in Norway. We collected the following data: Sociodemographic baseline data, surgical data, complication forms 3 and 12 months, Standing standardized coronal and sagittal radiographs, CT baseline, CT 12 months and Baseline MRI. Patient inclusion and inclusion criteria and collected data are displayed in Table 1.

**Table 1:** Patient inclusion and exclusion criteria for surgical and non-surgical treated patients

Inclusion criteria
1. Symptomatic Adult spinal deformity
2. At least 25 years of age
3. Oswestry disability score > 30
4. Additionally, one of the following:
• Scoliosis Cobb angle > 20
• Pelvic incidence Lumbar lordosis mismatch > 10
• C7 Sagittal vertical axis > 40 mm
• Pelvic tilt > 20
• Kyphotic fracture angel > 20
Exclusion criteria
1. Idiopathic scoliosis with adult progression, i.e. progression after skeletal maturity
2. Neuromuscular cause of deformity, diseases causing imbalance in the muscle strength and nerve control surrounding the spine
3. Acute fracture, < 3 months from injury
4. Malignancy, any cancer primary or metastasis of the spine

### Surgical Treatment (ST)

Surgical patients underwent deformity correction surgery at the surgeon’s discretion. Different surgical techniques were used (Table 3) with the goal of reducing scoliosis by > 50% and obtaining coronal (+/- 2 cm) and sagittal balance (+/- 4 cm).

### Physical exercises

A core strengthening and trunk stabilizing exercise program was developed by spine physiotherapists at Oslo University Hospital (Appendix 1). The program was available on the study homepage (intraks.org), and patients were referred to a physiotherapist for initial, and 3-month follow-up for instructions. They were supervised for the first three months, followed by structured homebased continuation. Intensity was progressively increased based on tolerance and symptom response. Training was documented in the patient log and a 12-month complication form. The choice of exercises was determined by the physiotherapists (level 1–3; light, medium, and hard), the ability of patients to perform the exercises 1–3 times a week, and to be continued for 12 months. Adherence was defined as completion of ≥ 75% of scheduled sessions during the 12 months. Detailed description of exercises, including sets and repetitions are provided in appendix 1. Additional training and physiotherapy outside the protocol was permitted. Analgesic medication was not standardized within the study protocol and was administered according to routine clinical practice. Although pain was not a formal exclusion criterion and participants were encouraged to continue training, symptoms severity may have influenced exercise tolerance and adherence. The absence of standardized analgesic management should therefore be considered when interpreting feasibility and adherence outcome. The exercise program was standardized across the study sites. We prospectively assessed feasibility and tolerability by recording adherence, completion rates, and predefined reasons for non-compliance or discontinuation of the exercise protocol.

### PROMS

PROMs were registered at baseline and 12-month follow-up using forms on the study homepage (intraks.org). The primary outcome parameter was the Oswestry Disability Index (ODI) [13–15], with the mean change in ODI from baseline to follow-up as the primary endpoint. Treatment success, defined as ≥ 30% improvement in ODI from baseline to follow-up, was a secondary outcome variable. Further secondary outcome parameters (1–4) were:

1. Clinical failure rate defined as ≥ 10% worsening in ODI.
2. Patient satisfaction evaluated using SRS-22 item 21 questionnaire (1 = very unsatisfied to 5 = very satisfied) [16].
3. Back and leg pain using the numeric rating scale (NRS) [17].
4. Reoperations registered 12-months post-surgery.

Treatment success was defined as a ≥ 30% improvement in ODI from baseline to 12 months. Although this threshold has not been specifically validated in adult spinal deformity populations, relative improvement thresholds are widely used in spine outcome research and may better account for differences in baseline disability than absolute change scores [18]. Given the substantial baseline disability in our cohort (mean ODI 49.9), we considered a 30% reduction to represent a clinically meaningful improvement.

### Complications

Complications were defined as any deviation from the expected postoperative or treatment course that required additional medical intervention, unplanned healthcare contact, or resulted in functional deterioration.

### Radiographs

Radiographs of the spine and pelvis, including the hip joints, were standardized and conducted at baseline and 12-month follow-up. Lumbar lordosis (LL), pelvic measurements, coronal Cobb angle, the C7 sagittal vertical axis (SVA), coronal balance, thoracic kyphosis, and Schwab type were quantified [19].

### Statistical analysis

Due to slower enrollment than anticipated, particularly during the COVID-19 pandemic, we ended the data collection before reaching the target number of patients as outlined in our protocol (ClinicalTrials identifier: NCT04536909). Continuous data are presented as mean (SD or 95% CI) or median (IQR), and categorical data as frequencies (%). The reported differences are between baseline and the last follow-up. Associations between two categorical variables were analyzed using the Pearson chi-squared test and the non-parametric Mann-Whitney U test was utilized for ordinal variables. Linear or Logistic regression methods were employed for three or more variables. Differences in means between the groups were analyzed using the Student’s t-test for two unpaired samples. A one-sample t-test was used to investigate the average change from baseline to final follow-up. Proportion of missing data in the primary outcome was approximately 10%. Missing values were assumed missing at random (MAR) and imputed using multiple imputations with linear regression methods. The imputed and complete case results are presented for comparison. Multiple linear regression was used for the continuous primary outcome (ODI change from baseline to 12 months). Multiple logistic regression was used for the categorical secondary outcome variable ( > = 30% improvement and < 30% improvement). Both models were applied to examine whether the between-groups difference depended on the baseline variables which were shown clinically and statistically significant.

The analysis was conducted with SPSS (version 29; IBM Corp., Armonk, NY, USA), and reported results adhere to the STROBE guidelines (strobe-statement.org). Further methods and covariates are shown in appendix 2.

## Results

**Fig. 1:**
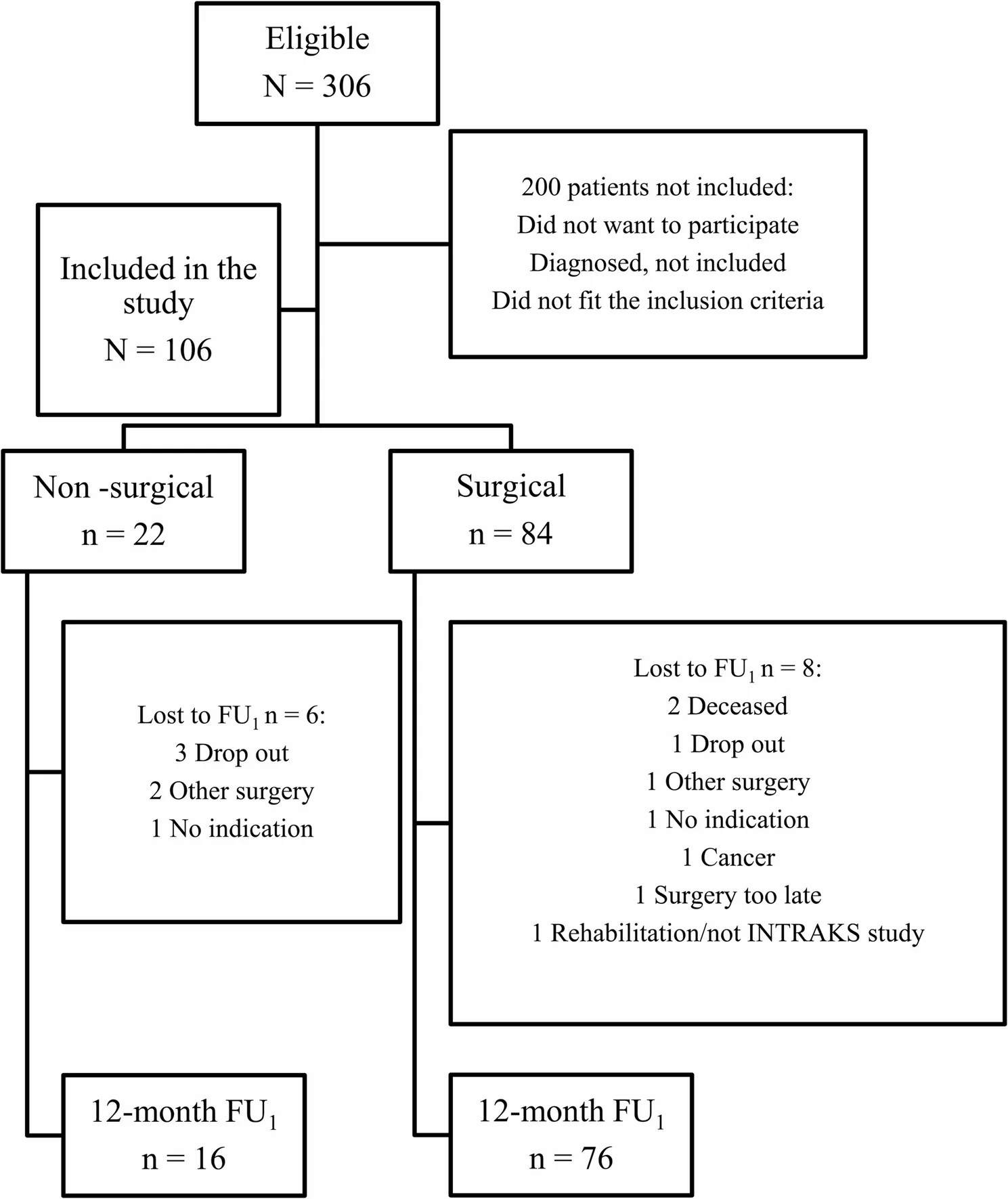
Flow chart for inclusion and _1_FU=follow up 2020 until 2024. No patients crossed over

A total of 76 surgical and 16 non-surgical patients were included in the study, with no crossover (Fig. 1).

The surgical group had a significantly higher baseline ODI than the non-surgical group (49.9 [SD = 17.5] vs. 35.1 [SD = 10.8]). There were no differences in the baseline characteristics of the Scandinavian and Japanese patients. Baseline characteristics are presented in Table 2, and surgical descriptive data in Table 3.

**Table 2:** Baseline data in surgical (*n* = 76) and non-surgical (*n* = 16) patients

Baseline variable	Surgical (SP)	Non-surgical (NSP)	*P*-value
	*n* (%)	*n* (%)	
Age, year, mean (SD_a_)	68.2 (8.2)	70.0 (9.3)	0.528
Female gender	45/71 (63.4)	14/16 (87.5)	0.064
Education			
No education	12/64 (18.8)	0	
< High School	25/64 (39.1)	8/15 (53.3)	
High School	14/64 (21.9)	3/15 (20.0)	
Graduate degree	14/64 (21.9)	4/15 (26.7)	
Work status			
100% Employed	7/70 (10.0)	1/15 (6.7)	
Part-time	2/70 (2.9)	1/15 (6.7)	
Retired	32/70 (45.7)	9/15 (60.0)	
Unemployed	12/70 (17.1)	0 (0.0)	
Partial sick leave	1/70 (1.4)	1/15 (6.7)	
Work Assessment allowance	2/70 (2.9)	1/15 (6.7)	
Disabled	12/70 (17.1)	2/15 (13.3)	
Smoking	9/70 (12.9)	0 (0.0)	0.132
Body mass index (kg/m2), mean (SD_a_)	25.3 (5.3)	26.4 (5.3)	0.430
ASA_b_			0.401
1	15/70 (21.4)	0 (0.0)	
2	38/70 (54.3)	13 (81.3)	
3	17/70 (24.3)	3 (18.8)	
Number of comorbidities, mean (SD_a_)	2.4 (1.9)	2.5 (1.1)	0.377
Depression/anxiety			0.213
Not depressed/anxious	27/70 (46.6)	0 (0.0)	
Somewhat depressed/anxious	23/70 (39.7)	8/14 (57.1)	
Very depressed/anxious	8/70 (23.8)	6/14 (42.9)	
Schwab Classification			
No coronal curves	50/66 (75.8)	7/16 (43.8)	
Lumbar coronal curve > 30 º	10/66 (15.1)	9/16 (56.3)	
Double curve thoracolumbar > 30 º	0	0	
PI-LL mismatch			0.011
< 10°	8/66 (12.1)	7/16 (43.8)	
10–20°	10/66 (15.1)	2/16 (12.5)	
> 20°	48/66 (72.7)	7/16 (43.8)	
Sagittal vertical axis (cm)			0.038
< 4 cm	6/66 (9.1)	6/16 (37.5)	
4–9.5 cm	22/66 (33.3)	4/16 (25.0)	
> 9.5 cm	38/66 (57.6)	6/16 (37.5)	
Pelvic tilt			0.364
< 20°	22/66 (33.3)	1/16 (6.3)	
20–30°	20/66 (30.3)	10/16 (62.5)	
> 30°	24/66 (36.4)	5/16 (31.3)	
ODI_c_, mean (SD_a_)	49.9 (17.5)	35.1 (10.8)	0.002
NRS_d_ Back pain, mean (SD_a_)	7.2 (1.9)	6.3 (3.1)	0.151
NRS_d_ Leg pain, mean (SD_a_)	5.0 (3.4)	3.8 (3.5)	0.235

*Abbreviations *: _a_*SD* Standard deviation, _b_ASA American Society of Anesthesiologists classification, _c_*ODI* Oswestry disability index, _d_*NRS* Numeric rating scale (pain)

**Table 3:** Surgical procedure types and surgery-specific variables in the surgical group (*n* = 76)

	Number of patients	%
	*n* = 72/76_a_	94.7%
Type of surgery		
Pedicle subtraction osteotomy (PSO)	37	51.4%
Lateral interbody fusion (LIF)	23	32.0%
Ponte Osteotomy	8	11.1%
Corpectomy/Vertebral column resection (VCR)	2	2.8%
Transforaminal lumbar interbody fusion (TLIF)	2	2.8%
Surgery specific variables		
Fused levels, mean (SD_b_)	7.5	2.6
Augmented screws	6	8.5%
Blood loss (ml)		
<500 ml	18	25.4%
500–999	23	32.4%
1000–1999	20	28.2%
≥2000	10	14.1%
Duration surgery (hours), mean (SD_b_)	6.4	2.0
Hospital stay (days), mean (SD_b_)	18.5	10.6
Cost of implant spine in Euro, mean (SD_b_)	11338.0	6950.3

*Abbreviations: *_a_Four patients undergoing surgery had missing surgical forms _b_*SD* Standard deviation

Degeneration was identified as the primary cause of spinal deformities among the surgically treated patients, accounting for 66 of 76 cases (86.8%). Additionally, 10 cases (13.2%) exhibited sequelae from spinal fractures resulting in surgically treated kyphotic deformities. The main reasons for spinal deformity in the non-surgical patients were (1) degeneration in 15/16 patients (94.0%) and (2) fracture sequela in 1/16 (6.0%). The customized training program and physiotherapy instructions were performed 1–6 times a week by 8 of 16 patients (50%); 8 of 16 (50%) patients failed to complete the exercises due to back or radicular pain. Additional physiotherapy was conducted in 11 of the 16 patients (69.0%). In total, 69% completed some form of exercise (customized training/physiotherapy). The final ODI in the non-adherent non-surgical group was 48.0 (SD = 11.2), and the adherent non-surgical group 36.2 (SD = 14.8).

### Primary outcome in surgical and non-surgical patients

The mean change in ODI for the surgical patients was an improvement of -16 (95% CI = -11.5, -20.5), from 49.9 (SD = 17.5) to 34.2 (SD = 20.5). In contrast, the non-surgical patients declined, with a mean ODI change of 7.0 (95% CI = -0.2, 14.2), deteriorating from 35.1 (10.8) to 42.1 (14.2) ODI points (Table 4).

**Table 4:** Primary and secondary outcome 0–12 months in surgical (ST; *n* = 76) and non-surgical (NST; *n* = 16) treated patients

Variable	Group	Baseline	12 months	Difference	95% CI	*n* (%)
		mean (SD_a_)/ frequency (%)				
Primary outcome						
ODI_d_	ST	49.9 (17.5)	34.2 (20.5)	-16.0 (18.5)	-20.5, -11.5	68/76 (89.5%)
	NST	35.1 (10.8)	42.1 (14.2)	7.0 (13.6)	-0.2, 14.2	16/16 (100%)
Secondary outcome						
Success_b_	ST		37/68 (54.4)		42.7, 65.7	68/76 (89.5%)
	NST		1/16 (6.3%)		1.1, 28.3	16/16 (100%)
Success MIx20_c_	ST		53.6%			
NRS_d_ Back pain	ST	7.2 (1.9)	3.8 (2.9)	-3.2 (3.4)	-4.1, -2.3	58/76 (76.3%)
	NST	6.3 (3.1)	7.3 (1.6)	1.0 (2.6)	-0.6, 2.6	12/16 (75.0%)
NRS_d_ Leg pain	ST	5.0 (3.4)	3.7 (3.1)	-1.2 (3.1)	-2.1, -0.3	50/76 (66.0%)
	NST	3.8 (3.5)	3.8 (3.1)	0.3 (2.6)	-1.4, 1.9	15/16 (94.0%)
SRS-22 satisfaction_e_	ST		58/76 (76.3%)			
	NST		14/16 (87.5%)			
Very satisfied	ST	NA	20 (34.5%)			
	NST	NA	0 (0%)			
Satisfied	ST	NA	13 (22.4%)			
	NST	NA	3 (25%)			
Neither satisfied nor unsatisfied	ST	NA	10 (17.2%)			
	NST	NA	6 (50%)			
Unsatisfied	ST	NA	8 (13.8%)			
	NST	NA	1 (8.3%)			
Very unsatisfied	ST	NA	7 (12.1%)			
	NST	NA	2 (16.7%)			

*Abbreviations: *_a_*SD*  Standard deviation _b_Success = ≥ 30% improvement in ODI _c_After x20 imputations and adjusting for baseline ODI difference and gender _d_*ODI* Oswestry disability index _d_*NRS* Numeric rating scale _e_*SRS-22 item 21*  Scoliosis research society 22 item 10: Are you satisfied with the result of your back management?

### Secondary outcomes in surgical and non-surgical patients

Secondary outcomes are displayed in Table 4. The treatment success rate was 54.4% (37 of 68) in the surgical patients and 6.3% (1 of 16) in the non-surgical patients. The response rate was 68/76 (89.5%).

We performed multiple imputation for ODI at baseline and 12 months, adjusting for baseline differences in ODI and gender. After imputation and adjustment, the treatment success rate was 53.6%.

The failure rate (≥ 10% worsening in ODI) was 13.2% in surgical patients and 50% for the non-surgical patients. Patient satisfaction was higher in the surgical group; half of the surgical patients expressed contentment. In the non-surgical group only one quarter achieved the same. NRS back pain improved by 3.2 points (95% CI = -4.1, -2.3) in the surgical group but worsened by 1.0 point (95% CI = -0.6, 2.6) in the non-surgical group. NRS leg pain showed minimal change in both groups.

Notably, 8 of 16 patients in the NST group were unable to complete the prescribed exercise program due to pain. Given the small sample size in the non-surgical group, feasibility and treatment failure outcomes should be interpreted as descriptive and exploratory.

When analysed as continuous ODI data, surgery was associated with greater ODI improvement relative to the non-surgical treatment (B = -23.0, 95% CI -32.8, -13.2, *p* < 0.001). In multivariate analysis adjusted for gender, baseline ODI, PI/LL mismatch and SVA after multiple imputation, surgery remained associated with greater improvement (B = -16.0, 95% CI -26.6, -5.4, *p* = 0.003) (Appendix 2, analysis A).

When analysed as binary ODI data at ≥ 30% improvement, 37/68 (54.4%) surgical patients achieved treatment success, and 1/16 (6.3%) in the non-surgical group. The crude odds ratio for improvement was 17.9 (95% CI 2.2, 143.3, *p* = 0.007). After adjustment for gender, baseline ODI, PI/LL mismatch and SVA after multiple imputation, surgery remains strongly associated with treatment success (adjusted OR = 25.3, 95% CI = 2.48, 259.27, *p* = 0.007) (Appendix 2, analysis B).

The continuous ODI analysis is likely more robust than the dichotomized responder analysis given the limited NST sample size.

### Surgical aspects

Pedicle subtraction osteotomy (PSO) [20] was used in 37/72 (51.4%) cases for deformity correction, followed by interbody cages such as lateral interbody fusion (LIF) 23/72 (32%) and trans foraminal interbody fusion (TLIF) 2/72 (2.8%). Estimated blood loss exceeded 1000 ml in 42.3% of patients, with 14.1% experiencing blood loss greater than 2000 ml. Further details are provided in Table 3.

### Complications and revision surgery

No complications were observed in the non-surgical patient group. Three of 76 surgical patients (3.9%) required revisions within 12 months due to 2 cases of proximal junctional kyphosis (2.6%) and 1 deep infection (1.3%). An additional 6 of 76 patients (7.9%) were identified as needing revision at 12-month follow-up, with a subsequent reoperation planned. These planned revisions were for proximal junctional kyphosis, screw loosening, dislocation of hooks, pseudarthrosis, back pain, and dislocation of set screws, each occurring in 1 out of 76 patients (1.3%). There were also three reports of paraparesis in the lower limbs 3/76 (3.9%), all following PSOs, classified as Frankel A, B, and C [21]. Complications are listed in Table 5.

**Table 5:** Complications and neurological deficits amongst surgical (*n* = 76) adult spinal deformity patients 0–12 months

	Surgical *n* (%)	Neurologic recovery status
Complication leading to re-operation diagnosed within first three months		
Proximal junctional kyphosis	1/76 (1.3%)	
Infection	1/76 (1.3%)	
Total complications reoperated 0–12 months	2/76 (2.6%)	
Neurological deficit diagnosed at primary hospital stay, no reoperation		No total recovery:
Peripheral nerve deficit_a_	4/76 (5.3%)	3 out of 4 improving
Paraparesis_b_		
Frankel_c_ A	1/76 (1.3%)	Improving
Frankel_c_ B	1/76 (1.3%)	Improving
Frankel_c_ C	1/76 (1.3%)	No improvement
Total neurological deficits	7/76 (9.2)	
Filed for revision at 12 months	1/76 (1.3%)	
Proximal junctional kyphosis	1/76 (1.3%)	
Screw loosening	1/76 (1.3%)	
Dislocation of hooks	1/76 (1.3%)	
Pseudarthrosis	1/76 (1.3%)	
Back pain	1/76 (1.3%)	
Dislocation of set screw	1/76 (1.3%)	
Total filed for revision at 12 months	7/76 (9.2%)	

*Abbreviations: *_a_Incomplete reporting of severity, defined 1–5 degree from light to sever. Two following Pedicle subtraction osteotomy, two after lateral combined with anterior interbody fusion. _b_all following Pedicle subtraction _c_Frankel classification for spinal cord injury ranging from normal senso-motoric function = E to no senso-motoric function = A

Table 5 List of complications and neurological deficits 0–12 months. There were no complications or neurological deficits amongst the non-surgical patients

### Radiographic measurements

Lumbar lordosis (LL) significantly increased from 19.5° to 40.2° after surgery (95% CI = 11.7, 34.5), and sacral slope (SS) also improved from 20.8° to 27.6° (95% CI = 5.8, 16.9). Correspondingly, C7-sagittal vertical axis (SVA) improved significantly from 12.7 cm preoperatively to 7.4 cm postoperatively (95% CI = -8.5, -0.1). Thoracic kyphosis (TK) increased from 31.0° to 44.2°, although this change was not statistically significant (95% CI = -0.1, 19.8). The Cobb angle showed a significant correction from 37.7° to 21.2° (95% CI = -30.3, -12.3). Details are included in Table 5. The predefined correction goals were achieved in a subset of patients: coronal balance was achieved in 37/55 (67.3%), sagittal balance 20/55 (36.4%), and a lumbar scoliosis (*n* = 30) was reduced by > 50% in 19/30 (63.3%). A high pelvic incidence (≥ 50°) was present in 36/66 (53%), and in 7/12 (58%) of the patients who underwent or were planning revision.

An increase in lumbar Cobb angle was observed in the NST group, with a mean difference of 4.6º (95% CI = 0.5, 8.7) between baseline and 12-month follow-up (Table 6).

Appendix 3 shows a comparison of patients with and without radiographic follow-up. The radiographs are most likely missing at random, yet findings are interpreted as exploratory as bias cannot be ruled out.

**Table 6:** Radiographs at baseline and 12-month follow-up with frequency of radiographs and independent sample t-test

Measurement	Group	Baseline	12 months	95% CI	*P*-value	Frequency/%
Lumbar lordosis, º	ST	19.5 (18.9)	40.2 (12.2)	11.7, 34.5	< 0.001	55/76 (72.4%)
	NST	43.3 (21.3)	42.4 (18.4)	-10.5, 4.0	0.350	13/16 (81.2%)
Pelvic incidence, º	ST	49.6 (12.1)	52.8 (10.8)	1.9, 14.0	0.011	54/76 (71.1%)
	NST	59.9 (14.4)	57.5 (16.2)	-10.5, 0.2	0.056	13/16 (81.2%)
Pelvic tilt, º	ST	25.8 (12.3)	25.0 (9.6)	-4.5, 8.5	0.545	54/76 (71.1%)
	NST	32.5 (13.3)	27.2 (6.9)	-7.8, 4.4	0.559	13/16 (81.2%)
Sacral slope, º	ST	20.8 (10.8)	27.6 (8.9)	5.8, 16.9	< 0.001	54/76 (71.1%)
	NST	32.1 (13.5)	29.3 (13.7)	-10.1, 0.2	0.057	13/16 (81.2%)
Cobb lumbar, º	ST	37.7 (16.0)	21.2 (13.8)	-30.3, -12.3	< 0.001	17/20 (85.0%)
	NST	36.0 (7.1)	40.7 (5.7)	0.5, 8.7	0.033	10/10 (100.0%)
Sagittal vertical axis, cm	ST	12.7 (8.4)	7.4 (5.6)	-8.5, -0.1	0.023	53/76 (70.0%)
	NST	6.8 (5.9)	6.4 (4.6)	-2.7, 1.6	0.592	13/16 (81.2%)
Coronal balance, cm	ST	2.8 (4.6)	1.9 (2.0)	0.8, -2.0	0.658	55/76 (72.3%)
	NST	2.6 (3.4)	2.8 (3.3)	-1.0, 1.4	0.767	13/16 (81.2%)
Thoracal kyphosis, º	ST	31.0 (22.5)	44.2 (15.2)	-0.1, 19.8	0.053	54/76 (71.1%)
	NST	43.3 (20.2)	43.6 (16.8)	-4.1, 8.3	0.481	13/16 (81.2%)

Table 6 Radiographs at baseline and 12-month follow-up with frequency of radiographs and independent sample t-test: 95% CI and *P*-value indicating the degree of change for the specific variables for surgical (ST; *n* = 76) and non-surgical (NST; *n* = 16) treated patients

## Discussion

This study included 76 surgical patients and 16 non-surgical patients. A statistically significant improvement in ODI was only observed amongst the ST patients: -16 (95% CI = -11.5, -20.5). Secondary outcomes were consistent with this result. In the surgical group, 37 of 68 patients (54.4%) achieved treatment success, defined as a ≥ 30% improvement in ODI. The non-surgical group showed an overall worsening of symptoms. The number of reoperations was low, with two cases of lower limb paraparesis observed following PSO. Incomplete correction of the deformity was also noted in some cases. Still, this study should be interpreted as an observational cohort analysis rather than a comparative effectiveness study. Given the non-randomized design, baseline imbalance, and differing recruitment settings between groups, between-group comparison is exploratory and hypothesis-generating. Because of the small NST sample size, the responder analysis and adjusted OR should be interpreted with caution.

We defined success as a ≥ 30% improvement in ODI. Although this threshold has not previously been used for adult deformity patients, it is widely applied in degenerative spinal disorders such as lumbar spinal stenosis and degenerative spondylolisthesis, and is considered to reflect clinically meaningful improvement more accurately than an absolute change score, as noted by Austevoll et al. [18]. Since patients in our cohort had substantial baseline disability, we considered a 30% reduction in ODI to represent a meaningful improvement from the patient’s perspective. Nielsen et al. defined a ≥ 20% improvement in ODI as a substantial clinical benefit in ASD patients aged ≥ 60 years and tracked outcomes at 1 week, 12 months, and 24 months [22]. Most ODI improvements occurred within the first year and were maintained at 24 months post-operatively, which is consistent with our 12-month results. Moreover, our baseline ODI was higher at 49.9, and 54.4% of surgical patients achieved a ≥ 30% improvement, surpassing Nielsen’s 45.5% success rate using a 20% threshold [22]. Stratification according to baseline ODI (< 40, 40–60, > 60) demonstrated an increasing likelihood of improvement with greater baseline disability (success rates of 33%, 50%, and 60%). The results in our study might differ if alternative ASD-specific thresholds are applied.

A key finding in this study is the limited feasibility of the NST protocol. Half of the patients were unable to complete the prescribed exercise program due to pain. This should not be interpreted solely as lack of treatment efficacy, but rather as a tolerability constraint that may limit the clinical application of this strategy in patients with ASD.

One factor in treatment selection is the risk of complications [2, 9]. No complications were observed in the non-surgical group; however, ODI worsened by an average of 28.8% from baseline to 12 months, indicating functional decline. In the surgical group, reoperation within 12 months occurred in only 3 of 76 patients (3.9%): two cases of proximal junctional kyphosis (PJK) and one deep infection. An additional six patients (7.9%) were scheduled for reoperation at 12-month follow-up. Postoperative neurological deficits occurred in 7 of 76 patients (9.2%), of which five had PSO procedures. Among these, three cases involved complete or incomplete paraparesis (Frankel A, B, C), with two cases (Frankel A and B) showing neurological improvement. None of the seven patients with neurological deficits required revision surgery.

The risk of neurological complications should not be underestimated. Three of the PSO cases (3.9%) had severe neurological deficits (Frankel A, B and C), with substantial impact on quality of life. Prior studies have reported neurological complication rates of 16.7% (72/423) and 11% (12/108) for patients undergoing PSO [9, 23], suggesting a consistent risk level.

NST showed limited efficacy in most patients, indicating that core strengthening and trunk stabilizing physiotherapy programs may be insufficient for ASD management. Despite less baseline deformity and lower ODI scores, the non-surgical group demonstrated very low treatment success. Half of the patients (8/16) were unable to perform the prescribed exercises due to pain, and progression of the lumbar Cobb angle was observed. Increased Cobb angle during follow-up may indicate that NST was insufficient for preventing structural deterioration. These findings suggest that, without adequate pain control, exercise-based therapy is not feasible for many patients, and not sufficient for preventing structural deterioration. Consequently, the deterioration in the NST group is most plausibly explained by natural disease progression in this symptomatic deformity cohort rather than by the exercise protocol itself. Additional physiotherapy outside the protocol was permitted to reflect routine clinical practice. Consequently, the study evaluates the feasibility of a structured exercise strategy implemented within usual care rather than the isolated efficacy of the protocol itself.

Few studies have directly compared ST and NST. In a multicenter prospective study by Kelley et al., ST showed superior outcomes compared to NST at both 12 and 24 months, regardless of randomization or observational grouping [8]. In their study, 77.4% of surgical patients (*n* = 142) achieved the MCID for ODI (10.9), compared to 38.3% in the non-surgical group (*n* = 144) [8]. Our own study aligns with these findings, suggesting that core strengthening and trunk stabilizing programs might not be enough for patients with significant disability.

In our cohort, some patients showed evidence of under correction, intentional or not. The target correction goals were achieved in 67.3% of patients for coronal balance (37/55), 36.4% for sagittal balance (20/55), and 63.3% for > 50% correction in lumbar scoliosis (19/30). Previous studies have reported that patients with a high PI are at increased risk for under correction [24]. We could not identify a similar pattern in our study. Proper spinal realignment is considered essential for achieving favorable clinical outcomes [25].

Although an increase in lumbar Cobb angle was observed in the NST group angle (mean + 4.6°, SD 5.8), this finding should be considered exploratory. The small sample size and limited radiographic follow-up preclude firm conclusions regarding structural progression or the ability of NST to prevent deformity progression. Still, this is consistent with findings from Kelley et al., who found that 25% of non-surgical patients achieved ≥ 5° correction of Cobb angle, relative to 99% in the surgical group [8].

Baseline differences between the surgical and non-surgical groups were notable. The mean baseline ODI was 49.9 (SD = 17.5) in the surgical group and 35.1 (SD = 10.8) in the non-surgical group. Female patients accounted for 63.4% (45/71) in the surgical group and 87.5% (14/16) in the non-surgical group (Table 2). The difference in baseline ODI was statistically significant (*p* = 0.002), and, while this limits direct comparison between groups, one would expect that patients in the non-surgical group — with lower disability — would have a higher capacity to participate in exercise (8/16, 50%) and physiotherapy (11/16, 69.0%). However, the treatment success rate was very low: only 1 of 16 patients (6.3%) achieved ≥ 30% ODI improvement, while 8 of 16 (50%) experienced ≥ 10% worsening. Patients with more severe deformity and greater disability were more likely to undergo surgery, reflecting both physician and patient preferences in clinical decision-making.

Regression to mean (rtm) may occur when there is a large difference/change from baseline to 12 months within a group without comparing with a control group. Therefore, we examined the change from baseline between the groups to avoid the problem with rtm and possibly adjust for clinically and statistically significant baseline difference between the groups. In the adjusted analysis after multiple imputation, surgery was associated with greater ODI improvement when analysed as a continuous outcome (Appendix 2). However, when the outcome was dichotomized at ≥ 30% improvement, the adjusted odds ratio was large with a wide confidence interval. The adjusted odds ratio should be interpreted cautiously because only one NST patient achieved treatment success, resulting in unstable estimates and wide confidence intervals. In this observational cohort, surgical treatment was associated with greater short-term improvement in disability relative to the non-surgical strategy implemented in this study; however, causal inference cannot be made. The small sample size precludes robust statistical comparison and limits the external validity of the NST results. The variable selection was guided by clinical relevance and prior literatures to avoid some problems like the risk of overfitting or unstable estimates since our sample size is limited.

### Limitations

The study is not randomized, and patient allocation was based on shared decision-making, introducing selection bias and limiting causal inference. The study population was recruited in three countries and includes Scandinavian and Japanese patients. We found no differences in their baseline characteristics; however, different ethnic backgrounds may influence how patients answer PROMs and evaluate satisfaction. Surgical indications and techniques may vary between countries, but the inclusion and exclusion criteria were the same. Furthermore, we had several discussions during the study period regarding techniques, which appeared to be similar. The number of NST patients is small, limiting a robust statistical comparison and limits the external validity of the NST results. Non-surgical patients were recruited only in Norway, potentially limiting generalizability. The COVID-19 pandemic substantially limited recruitment of NST patients in Sweden. In Japan, non-surgical treatment is largely managed within a private healthcare framework, which may impose a financial burden on patients and reduce participation. These factors contributed to the imbalance between treatment groups and should be considered when interpreting the NST results. Analgesic treatment was not standardized and was administered according to routine clinical practice. Given that a substantial proportion of NST patients did not complete the intervention due to pain, variability in pain management may have contributed to reduce adherence. The absence of a standardized analgesic protocol could therefore represent a confounding factor influencing both adherence and clinical outcomes in the NST group. Follow-up was limited to 12 months. As the planned sample size was not reached, especially in the non-surgical group, the risk of Type I and Type II errors, as well as residual bias, remains. The secondary outcome of ≥ 30% improvement in ODI has not prior been used for this type of patients but recommended in other degenerative spine diseases. The study was not designed or powered as a comparative effectiveness trial. The marked imbalance in group size, baseline disability, and recruitment settings (NST only in Norway) introduces substantial risk of residual confounding despite statistical adjustment. Therefore, between-group comparisons should be interpreted as exploratory and hypothesis-generating.

## Conclusion

A statistically significant improvement in ODI was only observed amongst the ST patients. Just over half of the surgical patients achieved successful treatment (≥ 30% improvement in ODI). A key finding was that 50% of NST patients were unable to adhere to the prescribed program due to pain. This suggests that feasibility and tolerability, rather than intrinsic inefficacy of core strengthening, may explain the limited short term clinical improvement observed. When choosing between a surgical and non-surgical approach for symptomatic ASD, it is essential to consider the risks of surgical complications as well as the likelihood of limited clinical improvement from NST. These findings should be interpreted within the context of a non-randomized observational design with baseline imbalance between groups. In conclusion, this study provides descriptive data on short-term outcomes following ST and highlights important feasibility limitations of the NST protocol. Comparative interpretation should be made with caution.

## Supplementary Information

Supplementary Material 1.

Supplementary Material 2.

Supplementary Material 3.

## Data Availability

The data in this paper is stored in “Service for sensitive data” (TSD) at the University of Oslo, and contains sensitive patient data ([https://www.uio.no/english/services/it/research/sensitive-data/index.html]).
