# Supplementary material for: Operative and non-operative treatment of symptomatic adult spinal deformity: a prospective multicenter observational study
Source: BMC Musculoskelet Disord. 2026 Jul 24;27:889. doi: 10.1186/s12891-026-10232-1 (PMC13681852; doi:10.1186/s12891-026-10232-1)
Supplement: Supplementary file 1 [file 12891_2026_10232_MOESM1_ESM.docx]

| **Appendix 1.0 Training and stretching program for the non-surgical patients, level 1-3, easy to hard** |  |  |  |
| --- | --- | --- | --- |
| E**xercise** | **Level 1** | **Level 2** | **Level 3** |
| Prone position shoulder lift, 3 sets, 10 reps | x |  |  |
| Supine position, thoracic mobilization with support below thoracic spine. Abduct the arm over the head, 3 sets, 10 reps | x |  |  |
| Supine position, stretch and lift the opposite arm and leg just above the ground, 3 sets, 10 reps | x |  |  |
| Supine position with soles on the floor, tilt the pelvis backwards, 3 sets, 10 reps | x |  |  |
| Supine position with legs flexed, lift the legs to 90 degrees in hips and knees: descend and up, one side at a time. 3 sets, 10 reps | x |  |  |
| Lie on the side with hands above the ears. Turn the upper body and hold for 30 sec, 5 sets. Repeat other side | x |  |  |
| Supine position, feet on the floor and lift the pelvis from the floor, tilt the pelvis backwards. 3 sets, 10 reps | x |  |  |
| Prone position, support below pelvis, lift one arm and opposite leg from the floor. 3 sets, 10 reps |  | x |  |
| Supine, feet on the floor and ball between legs. Lift the pelvis from the floor. 3 sets, 10 reps |  | x |  |
| Sitting position, straight legs and rubber band under feet. Pull the grip of the rubber band towards belly and back. 3 sets, 10 reps |  | x |  |
| Squat with chair: up and down until the buttocks almost touch the edge of the chair. 3 sets, 10 reps |  | x |  |
| Supine position, thoracic mobilization with support below thoracic spine. Fold hands behind head and press elbows together, 30 sec, 5 sets |  | x |  |
| Prone position, hands on the floor by your face and press upper body up as far as possible, hold for 1-2 sec, 30 sec, 5 sets |  | x |  |
| Reverse sit-up: sitting position, descend upper body towards floor. 3 sets, 10 reps |  | x |  |
| The cat: stand on all fours, inhale, sway back and look up, exhale, curve your back. 5-10 reps |  |  | x |
| Diagonal raise: stand on all fours and raise opposite leg and arm, keep stable and change sides. 3 sets, 10 reps |  |  | x |
| Dead bug: lie on back, 90 degree in hip and knee, arms elevated. Lower one arm and opposite leg. 3 sets, 10 reps |  |  | x |
| Thoracic extension: sitting upright, arms crossed, extend backwards, hold 1-2 sec. 3 sets, 5-10 reps |  |  | x |
| Lie on the side, knees bend, supported on one elbow, elevate pelvis until body is straight. 3 sets, 5-10 reps |  |  | x |
| The plank: hold the plank for 10-15 sec. 3 sets, 5-10 reps |  |  | x |
| Supine position, feet on floor, straighten one leg and straighten pelvis. 5 sets, 5-10 reps |  |  | x |
| Squat, bend knees until in a sitting position, press up again and repeat. 5 sets, 5-10 reps |  |  |  |
| **Stretching** |  |  |  |
| Hip extensors: lie on the back and hold both knees, let one drop towards the floor. Hold for 30 sec, 5 sets | x | x |  |
