# Supplementary material for: Operative and non-operative treatment of symptomatic adult spinal deformity: a prospective multicenter observational study
Source: BMC Musculoskelet Disord. 2026 Jul 24;27:889. doi: 10.1186/s12891-026-10232-1 (PMC13681852; doi:10.1186/s12891-026-10232-1)
Supplement: Supplementary file 2 [file 12891_2026_10232_MOESM2_ESM.docx]

| **Appendix 2 Unadjusted and adjusted binary and continuous ODI data** | | | | | | | | | | | | | | | | | | | | |
| --- | --- | --- | --- | --- | --- | --- | --- | --- | --- | --- | --- | --- | --- | --- | --- | --- | --- | --- | --- | --- |
|  | Original data | | | | | | | |  | Imputed data | | | | | | | | | | |
|  | Univariate analysis (N=84/92) | | | | Multivariate analysis ODI (N=84/92) | | | |  | Univariate analysis (N=92/92) | | | | Multivariate analysis (N=92/92) | | | | |  | |
| **A) Continuous ODI data (mean difference in ODI; Linear regression)** | | | | | | | | | | | | | | | | | | |  | |
|  | **B** | **95% CI** | **P-value** | **R-square** | **B** | **95% CI** | **P-value** | **R-square** |  | **B** | **95% CI** | **P-value** | **R-square** | **B** | | **95% CI** | | **P-value** | **R-square** | |
| Surgery, yes | -23.0 | -32.80, -13.20 | <0.001 | 0.21 | -15.68 | -26. 8, -4.60 | 0.006 | 0.32**_total_** | X | -23.0 | -32.64, -13.34 | <0.001 | 0.21 | -16.02 | | -26.61, -5.44 | | 0.003 | 0.33**_total_** | |
| Gender, female | 2.09 | -7.58, 11.80 | 0.668 | 0.002 | 1.80 | -7.01, 10.62 | 0.684 |  | X | 2.54 | -6.93, 12.02 | 0.599 | 0.004 | 2.72 | | -0.75, -11.19 | | 0.529 |  | |
| ODI baseline | -0.52 | -0.75, -0.30 | <0.001 | 0.20 | -0.31 | -0.56, -0.05 | 0.020 |  | X | -0.52 | -0.75, -0.30 | <0.001 | 0.20 | -0.36 | | -0.59, -0.12 | | 0.003 |  | |
| PI/LL mismatch | -0.24 | -0.45, -0.02 | 0.032 | 0.06 | -0.16 | -0.38, 0.06 | 0.144 |  | X | -0.22 | -0.43, -0.01 | 0.044 | 0.05 | -0.10 | | -0.30, 0.10 | | 0.340 |  | |
| Sagittal vertical axis | -0.65 | -1.18, -0.12 | 0.016 | 0.08 | -0.13 | -0.65, 0.40 | 0.633 |  | X | -0.67 | -1.18, -0.16 | 0.010 | 0.08 | -0.17 | | -0.66, 0.33 | | 0.507 |  | |
| **B) Binary ODI data (≥30% improvement in Oswestry Disability Index; Binary logistic regression)** | | | | | | | | | | | | | | | | | | | |  |
| **Covariates baseline** | **OR** | **95% CI** | **P-value** |  | **aOR** | **95% CI** | **P-value** |  | **MI_1_ x20** | **OR** | **95% CI** | **P-value** |  | **aOR** | **95% CI** | | **P-value** | | |  |
| Surgery, yes | 17.90 | 2.24, 143.27 | 0.007 |  | 22.82 | 2.23, 233.69 | 0.008 |  | X | 18.39 | 2.29, 147.50 | 0.006 |  | 25.35 | 2.48, 259.27 | | 0.006 | | |  |
| Gender, female | 0.74 | 0.28, 1.94 | 0.545 |  | 0.52 | 0.16, 1.58 | 0.248 |  | X | 0.74 | 0.28, 1.97 | 0.545 |  | 0.50 | 0.16, 1.53 | | 0.222 | | |  |
| ODI baseline | 1.02 | 0.99, 1.05 | 0.085 |  | 0.99 | 0.96, 1.02 | 0.673 |  | X | 1.02 | 1.00, 1.05 | 0.162 |  | 1.00 | 0.96, 1.00 | | 0.957 | | |  |
| PI/LL mismatch Schwab classification I | NA | NA | 0.302 |  | NA | NA | 0.254 |  | X | NA | NA | NA |  | 2.24 | NA | | NA | | |  |
| PI/LL mismatch Schwab classification II | 0.65 | 0.19, 2.19 | 0.487 |  | 2.00 | 0.39, 10.36 | 0.407 |  | X | 0.77 | 0.23, 2.56 | 0.665 |  | 0.53 | 0.43, 11.60 | | 0.335 | | |  |
| PI/LL mismatch Schwab classification III | 0.29 | 0.06, 1.51 | 0.142 |  | 0.32 | 0.06, 1.79 | 0.195 |  | X | 0.51 | 0.11, 2.27 | 0.373 |  | 1.05 | 0.11, 2.64 | | 0.433 | | |  |
| Sagittal vertical axis | 1.05 | 1.00, 1.12 | 0.201 |  | 1.05 | 0.98, 1.12 | 0.201 |  | X | 1.06 | 1.00, 1.13 | 0.047 |  | 0.86 | 0.98, 1.12 | | 0.201 | | |  |
| Hosmer and Lemeshow Test showed good fit for Logistic regression in all modalities | | | | | | | | | | | | | | | | | | | | |
| **12.8 ODI threshold; Logistic regression** | | | |  |  |  |  |  |  |  |  |  |  |  |  | |  | | |  |
|  | **OR** | **95% CI** | **P-value** |  |  |  |  |  |  |  |  |  |  |  |  | |  | | |  |
| Surgery, yes | 15.90 | 1.99 | 0.009 |  |  |  |  |  |  |  |  |  |  |  |  | |  | | |  |
| ODI baseline | 1.043 | 1.01, 1.07 | 0.004 |  |  |  |  |  |  |  |  |  |  |  |  | |  | | |  |
| **ODI baseline stratification** | | | | | **<40** | **40-60** | **>60** |  |  |  |  |  |  |  |  | |  | | |  |
| ≥30% improvement | |  |  |  | 33% | 50% | 60% |  |  |  |  |  |  |  |  | |  | | |  |
| 12.8 ODI treshold | |  |  |  | 25% | 50% | 65% |  |  |  |  |  |  |  |  | |  | | |  |
| _1_Multiple imputation _2_Relativ risk for success described by the covariate surgery in univariate and multivariate (same covariates as in the Logistic regression above) analyses using Poisson regression with log link and robust variance | | | | | | | | | | | | | | | | | | | | |

Table 1 Adjusted odds ratio (aOR) from multivariate logistic regression analysis for Success (≥30% improvement in Oswestry Disability Index), and a 12.8 ODI threshold as well as linear regression for mean difference in ODI in the original data and multiply imputed datasets (m = 20). ODI baseline stratification for success within the two thresholds.
