# Supplementary material for: Operative and non-operative treatment of symptomatic adult spinal deformity: a prospective multicenter observational study
Source: BMC Musculoskelet Disord. 2026 Jul 24;27:889. doi: 10.1186/s12891-026-10232-1 (PMC13681852; doi:10.1186/s12891-026-10232-1)
Supplement: Supplementary file 3 [file 12891_2026_10232_MOESM3_ESM.docx]

| **Appendix 3 Baseline data in patients with (n = 67) and without (n= 25) radiographic follow-up** | | | |
| --- | --- | --- | --- |
| **Baseline variable** | **With radiographic follow-up** | **Without radiographic follow up** |  |
|  | **n (%)** | **n (%)** |  |
| Age, year, mean (SD_1_) | 68.2 (7.7) | 69.2 (10.5) |  |
| Female gender | 42/66 (63.6) | 17/21 (81.0) |  |
| Education |  |  |  |
| Smoking | 6/66 (9.1) | 3/20 (15.0) |  |
| Body mass index (kg/m2), mean (SD_1_) | 25.3 (4.7) | 26.0 (5.2) |  |
| ASA_1_ |  |  |  |
| 1 | 11/67 (16.7) | 4/20 (20.0) |  |
| 2 | 38/67 (57.6) | 13/20 (65.0) |  |
| 3 | 17/67 (25.8) | 3/20 (15.0) |  |
| Number of comorbidities, mean (SD_1_) | 2.4 (1.9) | 2.3 (1.5) |  |
| **Schwab Classification** |  |  |  |
| Lumbar coronal curve >30 º | 17/67 (25.0) | 2/25 (8.0) |  |
| Double curve thoracolumbar >30 º | 0 | 0 |  |
| PI-LL mismatch |  |  |  |
| <10° | 13/67 (19.4) | 2/15 (13.3) |  |
| 10-20° | 11/67 (16.4) | 1/15 (6.7) |  |
| >20° | 43/67 (64.2) | 12/15 (80.0) |  |
| Sagittal vertical axis (cm) |  |  |  |
| <4cm | 11/67 (16.4) | 1/15 (6.7) |  |
| 4-9.5cm | 22/67 (32.8) | 4/15 (26.7) |  |
| >9.5cm | 34/67 (50.7) | 10/15 (66.7) |  |
| Pelvic tilt |  |  |  |
| <20° | 20/67 (29.9) | 3/15 (20.0) |  |
| 20-30° | 28/67 (41.8) | 2/15 (13.3) |  |
| >30° | 19/67 (28.4) | 10/15 (66.7) |  |
| ODI_2_, mean (SD_1_) | 46.9 (16.8) | 48.4 (10.8) |  |
| NRS_3_ Back pain, mean (SD_1_) | 7.1 (1.8) | 6.8 (2.5) |  |
| NRS_3_ Leg pain, mean (SD_1_) | 4.5 (3.3) | 5.6 (3.7) |  |
| Abbreviations: _1_SD = Standard deviation _2_ASA = American Society of Anesthesiologists classification _3_ODI = Oswestry disability index _4_NRS = Numeric rating scale (pain) | | | |
